# SSADH Variants Increase Susceptibility of U87 Cells to Mitochondrial Pro-Oxidant Insult

**DOI:** 10.3390/ijms21124374

**Published:** 2020-06-19

**Authors:** Giovanna Menduti, Alessandra Vitaliti, Concetta Rosa Capo, Daniele Lettieri-Barbato, Katia Aquilano, Patrizia Malaspina, Luisa Rossi

**Affiliations:** 1 Department of Biology, University of Rome Tor Vergata, 00133 Rome, Italy; giovanna.menduti@gmail.com (G.M.); vitalitialessandra93@gmail.com (A.V.); capo@uniroma2.it (C.R.C.); daniele.lettieri.barbato@uniroma2.it (D.L.-B.); katia.aquilano@uniroma2.it (K.A.); patrizia.malaspina@uniroma2.it (P.M.); 2 IRCCS Fondazione Santa Lucia, 00179 Rome, Italy

**Keywords:** GABA, SSADH, ALDH5A1, SSA, 4-HNE, paraquat, U87 cells, mitochondria

## Abstract

Succinate semialdehyde dehydrogenase (SSADH) is a mitochondrial enzyme, encoded by *ALDH5A1*, mainly involved in γ-aminobutyric acid (GABA) catabolism and energy supply of neuronal cells, possibly contributing to antioxidant defense. This study aimed to further investigate the antioxidant role of SSADH, and to verify if common SNPs of *ALDH5A1* may affect SSADH activity, stability, and mitochondrial function. In this study, we used U87 glioblastoma cells as they represent a glial cell line. These cells were transiently transfected with a cDNA construct simultaneously harboring three SNPs encoding for a triple mutant (TM) SSADH protein (p.G36R/p.H180Y/p.P182L) or with wild type (WT) cDNA. SSADH activity and protein level were measured. Cell viability, lipid peroxidation, mitochondrial morphology, membrane potential (ΔΨ), and protein markers of mitochondrial stress were evaluated upon Paraquat treatment, in TM and WT transfected cells. TM transfected cells show lower SSADH protein content and activity, fragmented mitochondria, higher levels of peroxidized lipids, and altered ΔΨ than WT transfected cells. Upon Paraquat treatment, TM cells show higher cell death, lipid peroxidation, 4-HNE protein adducts, and lower ΔΨ, than WT transfected cells. These results reinforce the hypothesis that SSADH contributes to cellular antioxidant defense; furthermore, common SNPs may produce unstable, less active SSADH, which could per se negatively affect mitochondrial function and, under oxidative stress conditions, fail to protect mitochondria.

## 1. Introduction

Succinic semialdehyde dehydrogenase (SSADH) is a mitochondrial homotetrameric enzyme, catalyzing a NAD^+^-dependent reaction in the catabolic pathway of γ-aminobutyric acid (GABA), the main inhibitory neurotransmitter of central nervous system. In order to avoid the persistence of its activity, GABA is cleared from the synaptic cleft mainly by the astrocytes and ends up in mitochondria where it undergoes two consecutive reactions. The first one, a transamination reaction, is catalyzed by GABA transaminase enzyme (GABA-T), leading to the formation of the succinic semialdehyde (SSA). In the second one, SSADH oxidizes SSA to succinate, thus supplying Krebs cycle, in the so called “GABA shunt” [1,2].

SSADH is translated as a 535 amino acid polypeptide, with the first 47 amino acids representing the mitochondrial signal peptide. Different mutations in the *ALDH5A1* gene (coding for SSADH) lead to enzyme failure and cause SSADH deficiency, a rare autosomal recessive disorder of childhood and the best characterized inherited metabolic disorder of GABA catabolism [1]. In the disease, the levels of GABA as well as of γ-hydroxybutyric acid (GHB) increase in cerebrospinal and other physiological fluids. Indeed, upon SSADH activity lacking, SSA is sidetracked to GHB by an alternative cytosolic reaction, catalyzed by an SSA reductase (SSAR). Both GABA and GHB are ultimately excreted with urine. When SSADH activity is missing, due to mutations of *ALDH5A1* gene, GHB increases, representing the pathognomonic feature of SSADH deficiency (therefore also called γ-hydroxybutyric aciduria).

Besides numerous pathological mutations, several common polymorphisms (SNPs) of the *ALDH5A1* gene also exist; some of them, when overexpressed in vitro, result in a decreased SSADH activity [3]. Recently, we demonstrated that a new mutation and a SNP, when present in combination in the same *ALDH5A1* allele, resulted in reduced SSADH activity upon in vitro overexpression. Furthermore, as demonstrated by in silico analyses, the level of mutated SSADH protein was decreased, likely due to tetramer instability and intracellular proteolysis [4,5].

It was demonstrated that SSADH displays an additional function consisting of the oxidation, and therefore detoxification, of 4-2-hydroxynonenal (4-HNE), a highly reactive and toxic byproduct of peroxidized polyunsaturated lipids that avidly binds to proteins [6]. Accordingly, SSADH knockout mice show increased lipid peroxidation and altered levels of antioxidants in various cerebral structures in association with mitochondrial damage and mitochondrial number and morphology alteration, leading to pexophagy and mitophagy [7,8].

In order to understand whether SSADH is involved in cell response to oxidative insult, in the present study we investigated the effects of *ALDH5A1* gene variants associated with lower enzymatic activity. In details, human U87 cells were transiently transfected with a cDNA construct harboring the alternative alleles for the three SNPs: c.106G > C, c.538C > T and c.545C > T. This CTT triple mutant (TM) encodes for a polypeptide showing three amino acid substitutions, i.e., p.G36R/p.H180Y/p.P182L, with the G36R mutation localizing in the mitochondrial signal peptide. We observed that TM protein level is lower with respect to that of the wild type (WT) protein, suggesting that TM may undergo protein degradation, concomitant to loss of enzyme activity. U87 cells expressing TM SSADH, when treated with the pro-oxidizing molecule Paraquat, show increased lipid peroxidation and accumulation of 4-HNE-protein-adducts. Mitochondrial damage also occurs as demonstrated by mitochondrial fragmentation and depolarization.

## 2. Results

### 2.1. Transient Overexpression of SSADH TM Mutant Results in Lower SSADH Protein Content and Enzyme Activity in U87 Cells

**Figure 1:**
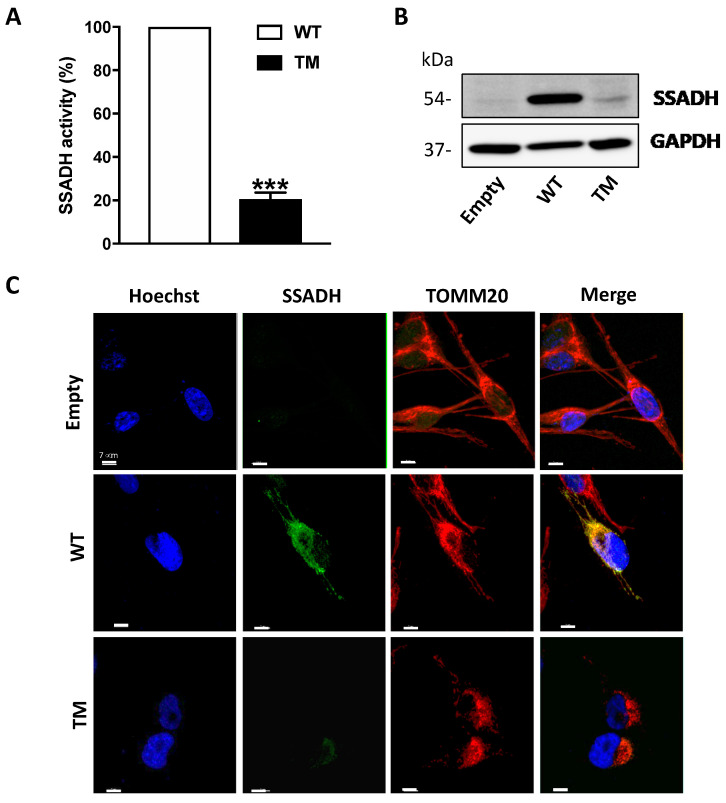
Enzyme activity and protein level of succinate semialdehyde dehydrogenase (SSADH) in U87 cells transfected with wild type (WT) and triple mutant (TM) constructs. U87 cells were transiently transfected for 24 h. (**A**) SSADH enzyme activity was assessed fluorimetrically. WT activity was considered as 100%. Data are expressed as mean ± SD of three separate assays from two separate transfections. *** *p* < 0.001, Student’s *t*-test. (**B**) Representative Western blot analysis of SSADH protein levels. Glyceraldehyde 3 phosphate dehydrogenase (GAPDH) was used as loading control. A total of 20 µg of total protein extract was loaded on each lane. (**C**) Confocal microscopy analyses of U87 cells immunostained for SSADH (green) and for the mitochondrial marker TOMM20 (red). Hoechst staining (blue) was used to label nuclei. Scale bar: 7 µm.

Transient overexpression of cDNA constructs harboring wild-type (WT) *ALDH5A1* gene, triple mutant (TM, c.106G > C, c.538C > T and c.545C > T, CTT), or the empty pcDNA3.1 vector (control) was performed in U87 glioblastoma cells. The enzymatic activity of SSADH was measured in total cell extracts by fluorimetric analysis and, as reported in Figure 1A, the amino acid replacements in the TM SSADH protein (p.G36R/p.H180Y/p.P182L) cause a strong decrease of enzyme activity (to about 20%) when compared to the WT protein. In line with this result, Western blot analysis performed on soluble fraction of cell lysates, revealed a strong decrease of the TM SSADH protein with respect to WT (Figure 1B). The higher level of WT SSADH protein when compared to the empty vector demonstrates the achievement of overexpression. Furthermore, Western blot analyses of Neomycin Phosphotransferase (NPT) protein, used as marker of transfection efficiency, revealed no difference between the three constructs (not shown).

In order to assess TM SSADH protein intracellular localization, confocal microscopy analyses of transfected U87 cells immunostained for SSADH (green) and for the mitochondrial marker TOMM20 (red) were carried out (Figure 1C). The results obtained demonstrate that both WT and TM SSADH localize in mitochondria, with the latter being in a very small amount, thus confirming the results obtained by Western blot analysis.

### 2.2. TM SSADH Decrease Does Not Depend on Proteasome-Mediated Degradation

**Figure 2:**
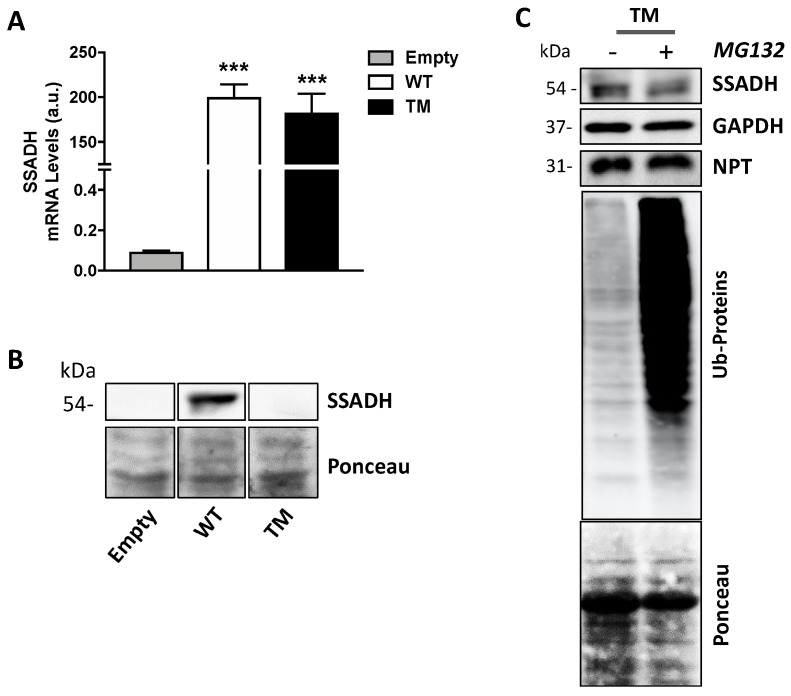
Triple mutant succinate semialdehyde dehydrogenase (TM SSADH) undergoes degradation in U87 cells. U87 cells were transiently transfected for 24 h. (**A**) RT-qPCR analysis of mRNA levels of ALDH5A1 gene, normalized for both β-Actin and NPT mRNAs. Data are shown as mean ± SD. *n* = 3, *** *p* < 0.001; (**B**) SSADH protein levels in the precipitated protein fraction. Ponceau staining was used as loading control. A total of 20 µg of total protein extract was loaded on each lane; (**C**) cells were treated with 10 µM MG132 proteasome inhibitor, during transfection, and TM SSADH and ubiquitinated proteins were assessed by Western blot analysis. Glyceraldehyde 3 phosphate dehydrogenase (GAPDH) was used as loading control, NPT for transfection efficiency, Ponceau protein staining as loading control for ubiquitinated proteins. Representative Western blot analyses are shown.

In order to investigate the mechanisms underlying the strong decrease in protein level and enzyme activity of TM SSADH, first we tested if such event could be attributed to reduced mRNA levels. To this end, RT-qPCR assays were performed on total RNA extracted from transfected U87 cells. The results in Figure 2A show no significant difference in the mRNA levels of TM SSADH with respect to WT. Proteins contained in the pellets obtained after centrifugation of the cell lysates were extracted and tested by immunoblotting, to rule out that TM SSADH protein was entrapped within the precipitated protein fraction, thus not revealed in Western blot shown previously in Figure 1B. A certain amount of WT SSADH protein was found in this insoluble fraction, possibly due to the high amount of WT protein present in the whole cell sample; however, no detectable TM SSADH protein was detected (Figure 2B). To investigate whether TM SSADH decrease was due to proteasome-mediated degradation, the proteasome inhibitor MG132 was added in culture medium concomitant to cell transfection and maintained for 24 h. Western blot analysis demonstrated that TM SSADH did not undergo proteasome-mediated degradation, as no change of its protein content was detected upon MG132 treatment. The accomplishment of proteasome inhibition was demonstrated by the significant accumulation of ubiquitinated proteins in cells treated with MG132 (Figure 2C). Given these results, degradation of the TM SSADH variant by proteasome can be ruled out.

### 2.3. TM SSADH Alters Mitochondrial Morphology and Increases Susceptibility to Oxidative Damage Induced by Paraquat

**Figure 3:**
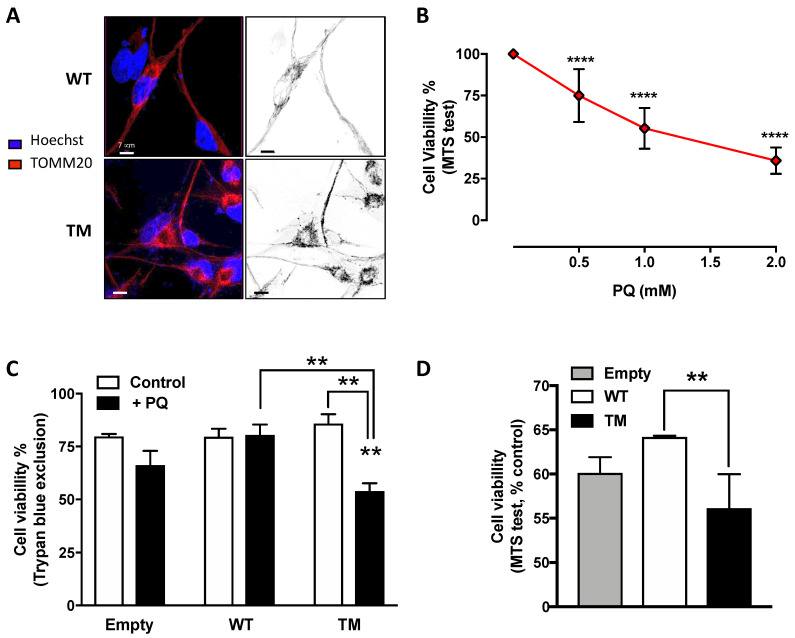
U87 cells transfected with triple mutant succinate semialdehyde dehydrogenase (TM SSADH) (TM SSADH) show mitochondrial fragmentation and increased susceptibility to the mitochondrial toxin Paraquat (PQ). (**A**) Mitochondrial network of untreated cells was pointed out by confocal microscopy by staining with the mitochondrial marker TOMM20 (red). Hoechst 33,342 (blue) was used to stain nuclei. Scale bar: 7 µm; (**B**) viability of non-transfected U87 cells was assessed by the MTS test, after 24 h treatment with increasing doses of PQ. Data are reported as percentage of cell viability of untreated cells and are shown as mean ± SD. *n* = 3, **** *p* < 0.0001; (**C**) viability of transiently-transfected cells was assessed by the Trypan blue exclusion test, after treatment with 1 mM PQ for 24 h (*n* = 3, ** *p* < 0.01). (**D**) Viability of transiently transfected cells was assessed by the MTS test, and treated as in (**C**) (*n* = 3, ** *p* < 0.01). Data are reported as percentage of cell viability of untreated cells and are shown as mean ± SD.

To unravel the effects of SSADH overexpression on mitochondria, we carried out confocal microscope analyses. Immunostaining using TOMM20 antibody revealed that mitochondria of cells overexpressing WT SSADH form tubular network-like structures throughout the cell body, whereas in cells expressing TM SSADH, the shape of the mitochondrial lattice appears fragmented (Figure 3A).

Given the observed mitochondrial morphology alteration and the possible implication of SSADH in the antioxidant defense, the effect of Paraquat (PQ), a mitochondrial toxin, was investigated in U87 cells overexpressing WT or TM SSADH. In preliminary experiments, non-transfected cells were treated with different PQ concentrations for 24 h and cell viability was assessed by MTS test, in order to select a suitable dose to be used in the experiments with the transfected cells. On the basis of the obtained results, we selected the dose of 1 mM as giving about 50% of cell death in control cells (Figure 3B). Hence, cell viability was assessed by Trypan blue exclusion test (Figure 3C) and by MTS test (Figure 3D) in U87 cells transfected with WT or TM SSADH constructs and treated with 1 mM PQ for 24 h. Both tests revealed increased susceptibility of cells transfected with the TM SSADH mutant to PQ treatment, with respect to cells transfected with WT SSADH.

**Figure 4:**
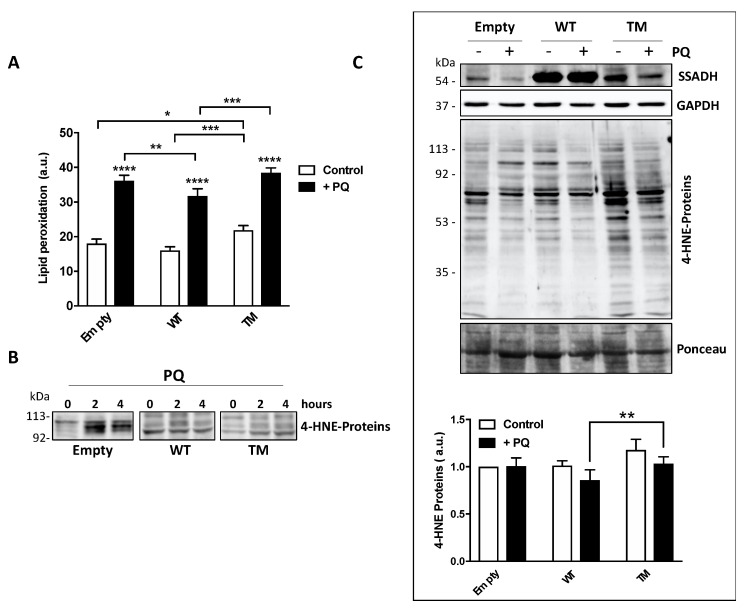
Markers of oxidative stress are increased in U87 cells transfected with the triple mutant succinate semialdehyde dehydrogenase (TM SSADH) upon paraquat (PQ) treatment. (**A**) Cells were transiently transfected for 24 h and then treated with 1 mM PQ. Lipid peroxidation was measured by C11 BODIPY using a flow cytometer. Ten thousand cells were analyzed for each sample and fluorescent intensities expressed as arbitrary units (counts within FL1-H fluorescence). Data are presented as mean ± SD. *n* = 3, * *p* < 0.05, ** *p* < 0.01, *** *p* < 0.001, **** *p* < 0.0001; (**B**) representative Western blot analysis of 4-HNE-modified protein levels in transfected cells treated with 1 mM PQ for 2 or 4 h. Immunoreactive bands with molecular weight around 100 kDa are shown. A total of 20 µg of total protein extract was loaded on each lane; (**C**) Western blot analysis of 4-HNE-modified protein levels upon 1 mM PQ treatment for 24 h. SSADH levels and glyceraldehyde 3 phosphate dehydrogenase (GAPDH) are also shown; (**D**) the intensity of the bands of the 4-HNE-modified protein levels in panel (**C**) was calculated with reference to both the untreated empty vector and protein staining with Ponceau. Data are expressed as mean ± SD, *n* = 4, ** *p* < 0.01.

PQ treatment induces peroxidation of membrane polyunsaturated fatty acids, which in turn are committed to degradation producing the protein reactive aldehyde 4-HNE, which represents an alternative substrate of SSADH [6]. Hence, lipid peroxidation rate was evaluated in U87 transfected cells either under basal conditions or upon PQ treatment by cytofluorimetric analysis prior staining with C11 BODIPY. PQ treatment increased the levels of lipid peroxides in all transfected cells (Figure 4A).

However, cells transfected with WT SSADH show a lower peroxidation level with respect to cells transfected with the empty vector; conversely, TM SSADH cells show higher level of lipid peroxides both upon PQ treatment and under basal conditions, suggesting that this mutant exerts a pro-oxidant action per se (Figure 4A). Upon lipid peroxidation, the formation of the degradation product 4-HNE occurs, and it covalently binds to proteins. After 2 or 4 h of PQ treatment, Western blot analysis, focused on the protein bands corresponding to 90–120 kDa, highlighted a significant accumulation of 4-HNE-protein adducts in cells transfected with the empty vector. Conversely, both WT SSADH and TM SSADH showed a limited accumulation of 4-HNE protein-adducts (Figure 4B). However, at longer time of PQ treatment (24 h), only cells transfected with TM SSADH show high levels of 4-HNE-protein adducts. This can be explained by previous observation that 4-HNE-protein adducts undergo removal within few hours [9]; however, the activity of TM SSADH is not sufficient to eliminate 4-HNE and the high level of protein adducts persists (Figure 4C).

### 2.4. TM SSADH Affects Mitochondrial Function and Increases Susceptibility of Mitochondria to Paraquat

**Figure 5:**
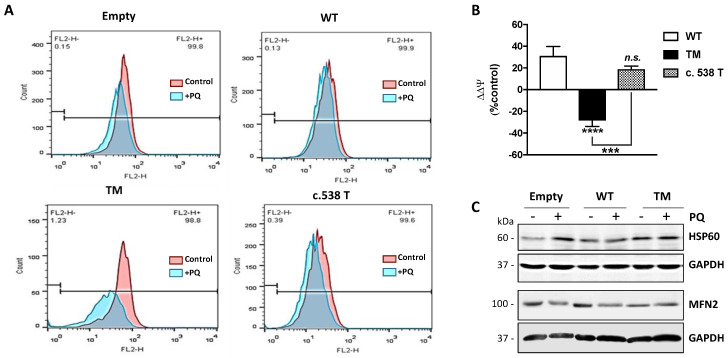
U87 cells transfected with triple mutant succinate semialdehyde dehydrogenase (TM SSADH) undergo mitochondrial damage upon paraquat (PQ) treatment. Cells were transfected for 24 h and then treated with 1 mM PQ for further 24 h. (**A**) Mitochondrial membrane potential (ΔΨ) was measured by cytofluorimetric analysis using Mitotracker Red staining. Representative profiles are reported from one representative experiment out of three. Fluorescence intensity of the probe was monitored as FL2-H (x axis, 599 nm) versus cell counts (y axis). Ten thousand cells were analyzed for each sample; (**B**) ΔΨ was normalized for mitochondrial mass (assessed by Mitotracker Green staining) and changes in ΔΨ (ΔΔΨ) after PQ treatment were represented as percentage with respect to cells transfected with the empty vector. Data are reported as mean ± SD. *n* = 3, *** *p* < 0.001, **** *p* < 0.0001. (**C**) Representative immunoblots of HSP60 and MFN2 protein levels. GAPDH was used as loading control. A total of 20 µg of total proteins was loaded on each lane.

Mitochondrial membrane potential (ΔΨ) is the driving force for ATP synthesis, and ΔΨ drop is indicative of impaired mitochondrial function [10]. We analyzed the effects of TM SSADH overexpression on mitochondrial function following 24 h PQ treatment by flow cytometry analysis using Mitotracker Red, a fluorescent dye specific for detecting ΔΨ (Figure 5A). In cells transfected with the empty vector, a slight mitochondrial membrane depolarization occurs, while WT SSADH transfected cells show almost no variation of ΔΨ. On the contrary, mitochondria of TM SSADH transfected cells strongly shift to a depolarization state, negatively affecting ΔΨ. Quantification of the changes of ΔΨ, carried out on replicated experiments, and normalized for mitochondria quantity (assessed by the ΔΨ-insensitive mitochondrial probe Mitotracker Green), confirmed these findings (Figure 5B). To further support these outcomes, we transfected U87 cells with the SSADH construct variant p.H180Y (c.538C > T), showing an in vitro enzyme activity similar to the WT SSADH [11]. As expected, these cells maintain ΔΨ at a level comparable to that of WT SSADH cells.

Collapsing of ΔΨ is generally associated with fragmentation of the mitochondrial network [12].

In order to better substantiate mitochondrial damage, we assessed protein levels of HSP60 and MFN2, which represent molecular markers of mitochondrial stress [13,14,15] and dynamics (fission/fusion), respectively (Figure 5C). Western blot analysis revealed that under resting conditions, the levels of Hsp60 in TM SSADH cells were greater with respect to control and WT SSADH cells and were maintained at high levels upon PQ treatment. By analyzing the protein levels of MFN2, which is implicated in mitochondrial fusion, we found that it was decreased in TM SSADH cells compared to both control and WT SSADH cells, confirming the occurrence of mitochondrial fragmentation under resting conditions. Upon PQ treatment, while MFN2 levels were reduced both in control and WT SSADH cells, in TM SSADH cells MFN2 levels remained unaltered, arguing that a maximum degree of mitochondrial fragmentation was already established upon TM SSADH overexpression.

## 3. Discussion

The accumulation of GABA catabolites is considered involved in the pathogenesis of many neuropsychiatric disorders [16,17] or neurodegenerative diseases, such as Alzheimer’s [2,18]. In particular, in SSADH deficiency, accumulation of GABA and its catabolite, i.e., GHB, is observed in physiological fluids; in association, impairments of GABAergic/glutamatergic neurotransmission, increase in oxidative stress as well as alterations in energy metabolism, autophagic processes and myelin are observed [7,8]. A possible role of GABA accumulation in inducing block of pexophagy and mitophagy has been also suggested [8]. Although the complete loss of SSADH activity has been deeply characterized, it remains unclear whether reduction of SSADH activity could contribute to the onset and progression of neuronal diseases and pathologies in which the GABAergic system is perturbed.

Therefore, in the present work, we studied the effects of variations in *ALDH5A1* gene (coding for SSADH) on oxidative stress response of a glial cell model (namely U87), focusing on mitochondrial damage, which is known to be associated with neuronal diseases [15,19,20,21,22,23]. We investigated the effects of transient overexpression of the TM SSADH (i.e., p.G36R/p.H180Y/p.P182L) in U87 cells. Indeed, the p.180Y/p.182L enzyme double variant has been shown to have an activity of 36% compared to the WT enzyme form [3]. However, it is not known whether the simultaneous presence of the p.36R change, which is located in the signal peptide addressing the protein to mitochondria, can also affect the SSADH enzyme activity or the stability and amount of the protein in the cell. Indeed, the TM variant represents one of the pathological alleles found in two different patients affected by SSADH deficiency who were heterozygous for another pathological mutation [3,24].

In the experiments carried out in this work, we showed that the expression of the TM variant leads to a strong reduction of both SSADH protein levels and enzyme activity compared to the WT form (to about 20%). No relevant difference in transfection and transcription efficiency was detected; therefore, we can hypothesize a lower stability of the TM protein compared to the WT one. This finding is not surprising as in previous work, it was demonstrated that proteins harboring double mutations could undergo destabilization of SSADH homotetramer, resulting in reduced protein content and activity [4,5].

We can envisage that the reduction of TM protein and activity might be the result of a synergistic effect caused by both structural alteration, due to the two changes in the amino acids p.180Y/p.182L in the mature peptide, and/or by a decreased mitochondrial import, since the p.36R change occurs in the mitochondrial leader peptide. However, the decrease of TM SSADH protein is not apparently caused by the proteolytic activity of the proteasome, since no recovery of SSADH protein content was observed upon its inhibition through MG132. Furthermore, we also excluded that TM SSADH protein could be entrapped within the precipitated pellet (cleared and not used for Western blot analysis). Actually, immunoblotting analyses of the insoluble fraction showed no presence of TM protein. Eventually, immunofluorescence analysis confirmed a decreased mitochondrial localization of the TM SSADH protein compared to the WT protein.

It was suggested that SSADH may play a role in antioxidant defense, primarily in brain tissue [6]. In order to consolidate this assumption in our system, we challenged the human glioblastoma cell line U87 overexpressing WT or TM SSADH with an oxidative insult, i.e., the bipyridyl herbicide paraquat (PQ), which is known to undergo redox cycling with O_2_, thereby producing reactive oxygen species. Indeed, PQ is considered as one of the risk factors for Parkinson’s disease, as it interacts with complex I of the mitochondrial electron transport chain and is toxic to cells [25]. Interestingly, cells overexpressing TM SSADH, upon PQ treatment, showed a significant reduction in cell viability compared to cells transfected with WT SSADH or with the empty vector. These data suggest that, under stress conditions, WT SSADH is protective, while the TM SSADH enzyme is not. Interestingly, we found that TM SSADH overexpression affects cell viability per se, and this is possibly due to affected import into mitochondria, or instability or noxious gain of function of the protein. These hypotheses are currently under investigation in our laboratory.

SSADH oxidizes, and therefore detoxifies, 4-HNE, a highly reactive and toxic degradation byproduct of peroxidized polyunsaturated lipids. SSADH acts specifically on 4-HNE in rat brain [6] and the accumulation of its derivatives has been found in brain tissues of the knockout mouse model for SSADH deficiency [7,26]. In order to corroborate the involvement of SSADH in the protection against lipid peroxidation, in this work we used sub-lethal doses of PQ not to provoke massive cell damage and found that TM SSADH cells show significantly higher lipid oxidation levels than both control and WT SSADH cells, either under basal conditions or after PQ treatment. On the other hand, WT SSADH transfected cells showed lower lipid peroxidation levels with respect to control cells, suggesting that overexpression of WT SSADH could help cells in counteracting oxidative damage.

Hill et al. [9] have shown that 4-HNE-modified proteins are rapidly removed by proteasomal and lysosomal pathways. In particular, most of the 4-HNE-modified protein bands (corresponding to 250, 150, 80, and 50 kDa) were removed within 3 h [9,27]. Therefore, accumulation of 4-HNE-modified proteins may represent an indication that cells undergo uncontrolled lipid peroxidation or prolonged impairment of processes removing damaged proteins. Our results showed that both WT SSADH and TM SSADH exert an early protection from the accumulation of 4-HNE-protein adducts. This is probably because the TM enzyme retains some activity, which may be sufficient to reduce the 4-HNE levels and its protein adducts at short time; this effect is no longer evident after 24 h PQ-exposure and thus 4-HNE-adducts accumulate.

In the mouse knockout model (*Aldh5a1*-/-), the absence of SSADH activity heavily alters mitochondrial function, particularly affecting oxidative phosphorylation, thus enhancing oxidative stress [28]. In our experimental model, following treatment with PQ, control cells showed a significant mitochondrial membrane depolarization, in terms of ΔΨ. Instead, cells transfected with WT SSADH buffered the drop of ΔΨ, suggesting that it has a key role in preserving the mitochondrial function during an oxidative insult. The same was observed in cells harboring the T allele for c.538C > T, which shows an enzyme activity similar to the WT enzyme. On the contrary, cells transfected with TM SSADH undergo a strong depolarization upon PQ treatment, indicating that TM SSADH is not able to protect mitochondrial function. Such different responses to PQ treatments did not depend on changes in the number of mitochondria, as the probing with Mitotracker Green, which measures the mitochondrial content and is insensitive to ΔΨ [29], did not reveal any significant variation (data not shown). These results provide evidence that reduced levels of SSADH activity could negatively affect mitochondrial function. Along this line, we revealed that mitochondrial network was significantly affected by TM SSADH overexpression as demonstrated by the occurrence of mitochondrial fragmentation and this could correlate with TM SSADH enzyme inability to maintain the ΔΨ and reduced state of lipids upon the PQ-mediated oxidative challenge. Further experiments are underway to deepen this aspect; other parameters of mitochondrial function, such as oxygen consumption, ATP production, expression and activity of the electron transport chain complexes, not exhaustively, will be measured in the future.

## 4. Materials and Methods

### 4.1. Construction of Recombinant ALDH5A1 cDNA Plasmids

The wild-type (WT) *ALDH5A1* cDNA (1605 bp), as well as the cDNA construct harboring the c.538T allele (c.538C > T) were cloned into pcDNA3.1 mammalian expression vector (Invitrogen, Waltham, MA, USA). To obtain the CTT construct (Triple Mutant, TM) for the c.106G > C, c.538C > T and c.545C > T variants, we used a double mutated construct harboring c.538T and c.545T alleles as template [11] to perform site-directed mutagenesis (Quick-Change Kit, Stratagene, La Jolla, CA, USA), according to manufacturer’s protocol. The following oligonucleotides were used to obtain the c.106C allele in PCR assay:

106C fwd 5’-GCCTGGTCCCTGCCTCCGGGCCTGCGCCCGGC-3’ and

106C rev 5’-GCCGGGCGCAGGCCCGGAGGCAGGGACCAGGC-3’

Sanger sequencing confirmed the correct mutagenesis and plasmid integrity. Purified plasmid DNA was used for subsequent transfections.

### 4.2. Cell Cultures and Transfections

The human glioblastoma cell line U87 was obtained by the American Type Culture Collection (ATCC, Manassas, VA, USA). Cells were cultured in DMEM-High Glucose (Sigma-Aldrich, St. Louis, MO, USA), supplemented with 10% FBS and penicillin/streptomycin (Lonza Sales, Basel, Switzerland). Cells were maintained at 37 °C under 5% CO2 atmosphere. A total of 24 h after plating, cells were transiently transfected with the cDNA constructs obtained by site-directed mutagenesis by using JetPEI transfection reagent (Polyplus, San Diego, CA, USA). The WT cDNA construct and the pcDNA3.1 empty vector were used as positive and negative control, respectively.

### 4.3. Quantitative PCR Analyses

Total RNA was extracted from transiently transfected cells using TRIzol Reagent (Invitrogen, Waltham, MA, USA). To evaluate possible differences in transfection efficiency between cDNA constructs, and ALDH5A1 mRNA amount and stability, total RNA (0.5 µg) was reverse transcribed using MMLV enzyme (Promega, Madison, WI, USA) with random primers. SYBR Green dye quantitative PCR was performed by using reverse transcribed cDNA (25 ng) and specific primer pairs for ALDH5A1, Neomycin Phosphotransferase II (NPT) and β-actin genes. Relative expression was evaluated using the 2-ΔΔCt method.

### 4.4. SSADH Activity Measurements of cDNA Mutant Constructs

Twenty-four hours after transfection, U87 cells were harvested with Trypsin-EDTA (Sigma-Aldrich, USA) solution, centrifuged 10 min at 700× *g*, washed, and resuspended in 100 mM Tris-HCl (pH 8.0). The cells were divided into two aliquots: two thirds were used to analyze SSADH enzyme activity and protein levels and one third to extract total RNA to evaluate transfection and transcription efficiency. The samples were sonicated, and protein concentration was determined by the Lowry method (BioRad, Hercules, CA, USA). SSADH activity was measured fluorimetrically, by using SSA and NAD^+^ as substrates and monitoring NADH fluorescence, excitation 355 nm, emission 470 nm (Perkin Elmer LS 50/B fluorimeter, Waltham, MA, USA), and expressed as nmol NADH/min per mg of total protein. The enzyme activity was normalized according to the transfection efficiency obtained for each cDNA construct [4,5].

### 4.5. Cell Treatments

Transfected or untransfected U87 cells were seeded at density of 4 × 104/cm^2^. Paraquat (PQ) (Methyl viologen dichloride hydrate, Sigma-Aldrich, St. Louis, MO, USA) was dissolved in H_2_O and administered to cells after 24 h from transfection. The proteasome inhibitor MG132 (Abcam, Cambridge, UK) was dissolved in DMSO and added to the cells simultaneously with cell transfection (final concentration 10 μM for 24 h).

### 4.6. Cell Viability Assays

After PQ treatment cell viability was determined by Trypan Blue dye exclusion assay (0.2% *w*/*v*) (Sigma-Aldrich, St. Louis, MO, USA) by a phase contrast microscope. For the MTS assay, U87 cells were seeded into 96-well plates at a density of 10,000 cells per well (in 200 µL complete medium). The CellTiter 96^®^ AQueous One Solution Cell Proliferation Assay kit (Promega, Madison, WI, USA) was added, following the manufacturer’s instructions and cells were incubated at 37 °C for 2–4 h. Absorbance was detected at 490 nm by a microplate reader (Sunrise Tecan Microplate Reader, Mannendorf, Switzerland).

### 4.7. Western Blot Analysis

Cells were harvested by Trypsin-EDTA solution, centrifuged 10 min at 700× *g*, washed, and suspended in 100 mM Tris-HCl (pH 8.0). After sonication, an equal amount of 2× lysis buffer (150 mM NaCl, 50 mM Hepes, 1% *v/v* Triton X-100, 1 mM EDTA, 1% *v/v* NP-40) supplemented with Protease Inhibitor Cocktail (Sigma-Aldrich, St. Louis, MO, USA) was added and the samples were incubated on ice for 30 min.

Lysates were then centrifuged at 12,000× *g* at 4 °C for 30 min and total protein content of the supernatants assessed. Samples were diluted in 2× Laemmli buffer and boiled at 95 °C. Aliquots of the denatured samples underwent 10% SDS-PAGE. Molecular weight markers (AccuRuler RGB Prestained Protein, Maestrogen, Hsinchu City, Taiwan) were loaded on separate lanes. Proteins were transferred onto a nitrocellulose membrane by a Trans-Blot^®^ Turbo™ transfer System (Bio-Rad, Hercules, CA, USA). Ponceau Staining (Bio-Rad, Hercules, CA, USA) of the membranes was used as loading and transfer control. Upon saturation, membranes were incubated with two different mouse monoclonal primary anti-SSADH antibodies (epitope 142–163; #sc-515022 and epitope 1–226; #sc-390754 from Santa Cruz Biotechnology, Dallas, TX, USA). Rabbit anti-Neomycin Phosphotransferase II polyclonal antibody (NPT, used to assess transfection efficiency), and goat anti-4-HNE polyclonal antibody (to detect 4-HNE-protein adducts) were from Millipore, Burlington, MA, USA. Furthermore, mouse anti-GAPDH monoclonal antibody and mouse anti-ubiquitin monoclonal antibody were from Sigma-Aldrich, St. Louis, MO, USA. Monoclonal anti-HSP60 and anti-MFN2 antibodies were from Abcam (Cambridge, UK). Secondary antibodies were from Santa Cruz Biotechnology (Dallas, TX, USA). Chemiluminescent detection was performed using Clarity™ Western ECL Blotting Substrates (Bio-Rad, Hercules, CA, USA) and the Fujifilm Las-3000 imaging system (Fuji, Tokyo, Japan). Densitometric analysis was performed by using the ImageJ software (NIH, Bethesda, MD, USA).

### 4.8. Measurement of Lipid Peroxidation

PQ-treated or untreated U87 cells were incubated for 30 min at 37 °C with 10 μM Lipid Peroxidation Sensor-fluorescent probe BODIPY™ 581/591 C11 (Thermo Fisher Scientific, Waltham, MA, USA). The fluorescence intensity of 10,000 stained cells from each sample were analyzed by FACScalibur instrument (Becton-Dickinson, Franklin Lakes, NJ, USA). Data were analyzed using FlowJo™ software (TreeStar; Ashland, OR).

### 4.9. Assay of Mitochondrial Membrane Potential

After PQ treatment, cells were incubated with 250 nM MitoTracker Red and 250 nM Mitotracker Green (Life Technologies Ltd., Waltham, MA, USA) for 30 min, at 37 °C. After washing, cytofluorimetric analysis of samples was performed by cytofluorimetry. Ten thousand cells were analyzed for each sample. Channel FL2-H (585/42) and FL1-H (530/30) were utilized for the detection of Mitotracker Red and Mitotracker Green, respectively. Mitochondrial membrane potential (ΔΨ) was normalized to mitochondrial mass using Mitotracker Green staining and changes in ΔΨ (ΔΔΨ) were expressed as percentage with respect to cells transfected with the empty vector. Data were analyzed using FlowJo™ software (TreeStar; Ashland, OR).

### 4.10. Immunofluorescence Analysis

U87 cells were grown on coverslips and treated with PQ, washed three times in PBS (Phosphate Buffered Saline: 140 mM NaCl; 2.7 mM KCl; 8 mM Na_2_HPO_4_; 1.8 mM KH_2_PO_4_) and fixed with 4% paraformaldehyde for 10 min at room temperature. Cells were then permeabilized with PBS/Triton X-100, 0.4% (*v*/*v*), blocked for 1 h with PBS/FBS 10% (*v*/*v*), and incubated overnight with the following primary antibodies: mouse anti-SSADH monoclonal antibody (1:50, #sc-390754 Santa Cruz Biotechnology, Dallas, TX, USA) and rabbit anti-TOMM20 polyclonal antibody (1:200, Santa Cruz Biotechnology, Dallas, TX, USA). Cells were then washed with PBS and incubated for 1 h with fluorophore-conjugated host-specific secondary antibodies: goat anti-mouse Alexa Fluor 488 and goat anti-rabbit Alexa Fluor 555 secondary antibody (Thermo Fisher Scientific, Waltham, MA, USA). Nuclei were stained by incubating cells with 1 μg/mL Hoechst 33,342 dye (Thermo Fisher Scientific, Waltham, MA, USA) in PBS, 15 min at room temperature. Cell fluorescence was detected with confocal microscopy using Olympus Fluoview 1000 Confocal Laser Scanning System (Olympus, Tokyo, Japan), equipped with an Olympus IX-81 inverted microscope. A 63× magnification oil immersion objective (NA 1.42, WD 0.15 mm) was used for all image acquisitions using ImageJ software (NIH, Bethesda, MD, USA).

### 4.11. Statistical Analysis

The results are presented as means ± S.D. One-way ANOVA, with Turkey multiple comparison Test, and PRISM 6 (GraphPad Prism software, San Diego, CA, USA), or unpaired Student’s *t*-test were used, as indicated.

## 5. Conclusions

In conclusion, the results of the present work demonstrate that in vitro expression of TM SSADH produces a reduction of cell viability and mitochondria damage per se. Furthermore, we confirmed that SSADH participates in antioxidant defense, and its decreased activity impairs cell capability to cope with oxidative stress. These effects may also be exacerbated by an altered intermediate cell metabolism and cell ATP production, since SSADH takes part in the GABA shunt, producing succinate (which fuels Krebs’ cycle) as well as NADH [30]. Our findings could be also relevant for the impact of this SSADH variant in the general population. The frequency of TM haplotype is relatively low in Europeans (4.3% in the Italian population, Ciminelli et al. JAD, in press), and therefore the occurrence of a SSADH tetramer formed exclusively by TM subunits (as in this in vitro study) is very rare. However, it may occur in heterozygosity with the alternative haplotypes GCC or GTC; producing different combinations of the subunits in the SSADH tetramer with suboptimal activity, possibly responsible for subclinical conditions.
